# Directions of Longitudinal Relationships between Housing-related Control Beliefs and Activities of Daily Living among People with Parkinson’s disease

**DOI:** 10.1177/23337214241299084

**Published:** 2024-11-11

**Authors:** Nilla Andersson, Susanne Iwarsson, Susann Ullén, Björn Slaug, Maria H. Nilsson

**Affiliations:** 1 Department of Health Sciences, Lund University, Sweden; 2 Clinical Studies Sweden – Forum south, Skånes University Hospital, Lund, Sweden; 3 Clinical Memory Research Unit, Department of Clinical Sciences Malmö, Lund University, Lund, Sweden; 4 Memory Clinic, Skåne University Hospital, Malmö, Sweden

**Keywords:** neurological disease, perceived housing, daily activities, environmental gerontology

## Abstract

**Introduction:**

The gerontological literature suggests that external housing-related control beliefs (HCB) influence activities of daily living (ADL) among older people, but knowledge is scarce for people with Parkinson’s disease (PD). This longitudinal study aimed to explore the directions of the relationship between external HCB and ADL among people with PD.

**Methods:**

Baseline (T1) and 3-year follow-up data (T2) were collected from 154 people with PD (mean age = 68 years, T1). Two regression analyses were applied, where dependent (T2 values) and independent (T1 values) variables—external HCB score and PD specific ADL (PADLS)—were switched, adjusting for age, disease severity, cognitive functioning, and accessibility problems.

**Results:**

There was a significant effect of ADL on external HCB (β = 3.07, *p* < .001, CI [1.28, 4.85]), but no effect in the reverse direction. The proportion with moderate-extreme ADL difficulties increased over time (from 20.8% to 32.5%, *p* = .006).

**Discussion:**

ADL difficulties seem to lead to higher external HCB, but not the other way around, which contradicts assumptions in environmental gerontology theories. This new knowledge can promote theory development. While additional studies are required to verify whether this is a disease-specific finding, this indicates the importance of targeting ADL if the purpose is to influence external HCB among people with PD.

## Introduction

Parkinson’s disease (PD) is a progressive disease with symptoms that deteriorate over time. The prevalence is 1% to 2% for people over 65 years (Váradi, 2020), and the PD population is expected to double by 2040 (Dorsey et al., 2018). With the characteristic motor and non-motor symptoms of the disease (Váradi, 2020), activities of daily living (ADL) can already be negatively affected when a person is diagnosed (Hariz & Forsgren, 2011). ADL includes fundamental skills needed for the patient to care for themselves in daily activities, covering both personal and instrumental activities (Mlinac & Feng, 2016). Most ADL domains deteriorate over time for people with PD (Sperens et al., 2020), and a study showed that people with self-reported PD are more dependent in ADL than very old adults in the general population (Nilsson et al., 2013). Autonomy and independence have been considered to relate to perceived control (Skinner, 1995), and the sense of being in control in the immediate living environment is important (Schulz & Heckhausen, 1999).

Perceived control of the home has been conceptualized as housing-related control beliefs (HCB) (Oswald et al., 2003) and is related to person-environment (P-E) exchange (Lawton & Nahemow, 1973). HCB addresses how people think they can deal with daily issues related to their home (Oswald et al., 2003). Focusing on external control beliefs in the current study, this dimension addresses whether a person relies upon external influences in relation to the home, such as powerful others, chance, or faith (Oswald et al., 2003; 2006). It has been suggested that processes involving control beliefs in relation to the home change with age (Chaudhury & Oswald, 2019). This has been empirically supported by a study reporting that very old people have higher external HCB than younger older adults (Ekström et al., 2016). Cross-sectional studies have shown that higher external control beliefs are associated with lower life satisfaction, more depressive symptoms, and lower psychological well-being among older people in general (Kylén et al., 2017; Oswald, Wahl, Schilling, Nygren, et al., 2007; Wahl et al., 2009).

Cross-sectional studies among very old people have shown that higher external HCB is associated with ADL dependence (Iwarsson et al., 2007; Oswald, Wahl, Schilling, & Iwarsson, 2007; Tomsone et al., 2013). In younger older people (67–70 years), external HCB have been shown to have a mediating effect on the relationship between housing accessibility and ADL (Gefenaite, Björk et al., 2020). The relationship between external HCB and ADL is therefore dynamic and could be specific for different sub-groups of the aging population. For example, the relationship could be different among people aging with a progressive disease. As people with PD often have difficulties in ADL (Sperens et al., 2020) and a sense of gradually losing control due to the disease (Sjödahl Hammarlund et al., 2018), knowledge about the relationship between ADL and external HCB could add to the overall understanding of housing and health interactions for people aging with PD.

A recent literature review highlighted a substantial knowledge gap regarding people with PD and housing issues (Iwarsson et al., 2022). One study identified in the review addressed the cross-sectional relationship between external HCB and ADL among people with PD, suggesting that more difficulties in ADL were associated with higher external control in relation to the home (Nilsson et al., 2016). A longitudinal study suggested that, while housing accessibility problems predicted increased ADL difficulties over time, external HCB moderated that effect (Gefenaite, Björk et al., 2020). This points to a complex interaction that can be unique for different sub-groups.

Theoretical frameworks addressing the general aging population have assumed that negative perceived aspects of housing (such as higher external HCB) lead to health problems over time (Chaudhury & Oswald, 2019; Oswald et al., 2006; Wahl et al., 2012). Previous research has not longitudinally explored both directions of the relationships between external HCB and ADL. Moreover, having a disease like PD where ADL is affected early, the direction of the relationship might be different. Therefore, the aim of this study was to explore the directions of the relationship between external HCB and ADL among people with PD, with specific attention to changes over time. Specifically, we explored these two hypotheses:

1. More difficulties in ADL lead to higher external HCB;
2. Higher external HCB lead to more difficulties in ADL.

## Methods

This is a cohort study, with data derived from the longitudinal cohort study “Home and health in people aging with Parkinson’s disease” (HHPD; Nilsson & Iwarsson, 2013). The baseline assessment was conducted in 2013 and the 3-year follow-up in 2016 (Nilsson & Iwarsson, 2013). To explore the directions of the relationship between external HCB and ADL two regression analyses were applied, where the variables used as dependent and independent were switched.

## Data Collection

Data were collected through a self-administered postal survey and a subsequent home visit. The home visit included interview-administered questionnaires, observations, and clinical assessments. All data collectors were experienced health care professionals who underwent project-specific training, that is, education and practical training on how to conduct the data collection and scoring; for example, a specific course focusing on the Housing Enabler (HE) instrument. Data were initially collected working in pairs, with one person conducting the interview and clinical assessment while the other observed and listened, and regular reconciliation discussions were held. The HHPD was approved by the Regional Ethical Review Board in Lund (Nos. 2012/558 and 2015/611). The study was conducted in accordance with the principles of the Helsinki Declaration and written informed consent was collected for each participant.

## Recruitment and Participants

The HHPD project recruited participants from three hospitals in Skåne County, Sweden. The inclusion criterion was being diagnosed with PD for at least 1 year, and exclusion criteria were difficulties in understanding or speaking Swedish (*n* = 10), severe cognitive difficulties (*n* = 9), living outside Skåne County (*n* = 58), or other reasons (*n* = 57). The final baseline sample was *N* = 255, and all were potential participants at the 3-year follow-up data collection. By then 22 participants were deceased, three had moved, and one was outside the 3 years ± 3 months follow-up window. Consequently, 229 people were eligible and invited to participate. Of those, eight were not reachable, four had their diagnosis revised, 51 declined, and one showed extensive missing data, leaving us with *N* = 165. As we included only participants with data for the main variables at both data collections, the total sample was *N* = 154. At baseline, the mean age (min-max) was 68.1 (45–88) years with a mean (min-max) PD duration of 9.3 (2–43) years; 52 (33.8%) were women (Table 1).

**Table 1.** Sample Characteristics (*N* = 154).

Variable, n (%) unless stated otherwise	T1	T2	Missing, *n*
	*n* (%) unless stated otherwise		(T1/T2)
Sex, men	102 (66.2)	102 (66.2)	-
Age, mean (SD)	68.1 (8.8)	71.2 (8.8)	-
PD duration (years), mean (SD)	9.3 (6.4)	12.3 (6.4)	-
Motor symptoms (UPDRS III), median (q1–q3)	28 (21–37)	27 (20–38)	0/10
Mobility device (indoor)			
Any mobility device	40 (26.3)	84 (60.4)	1/7
Walking stick/crutches	17 (11.0)	28 (18.2)	0/0
Walking frame, walking table, quad-based walking stick	3 (2.0)	9 (5.8)	1/0
Rollators	16 (10.4)	34 (22.4)	0/2
Manual wheelchair	3 (1.9)	11 (7.2)	0/2
Powered wheelchair	1 (0.6)	2 (1.3)	0/4
Higher education (university)	57 (37.0)	-	-
Living with a partner	121 (78.6)	117 (76.0)	-
Type of housing			-
Apartment	59 (38.3)	64 (41.6)	
One-family house	92 (59.7)	87 (56.5)	
Other	3 (1.9)^a^	3 (1.9)	
Residential location			-
Rural	52 (33.8)	56 (36.4)	
Semi-urban	37 (24.0)	33 (21.4)	
Urban	65 (42.2)	65 (42.2)	
Housing tenure			
Privately owned	122 (79.2)	119 (77.3)	-
Rental	32 (20.8)	35 (22.7)	-
Years in present dwelling, mean^b^ (SD)	21.3 (16.3)	21.9 (16.1)	-
Accessibility problems (HE score), median (q1–q3)	166 (80.5–239.5)	234 (120.0–353.0)	0/1

*Note.* T1 = baseline; T2 = 3-year follow-up; PD = Parkinson’s disease; UPDRS = Unified Parkinson’s Disease Rating Scale, part III; HE score = Housing Enabler total score.

a Due to rounding of decimals, the percentage of “Type of housing” is not 100%.

b Information provided by participants in interviews. Seventeen participants moved to other dwellings between T1 and T2.

## Variables

### External Housing-Related Control Beliefs

We used the external Housing-Related Control Beliefs Questionnaire, which addresses whether chance/luck or help from others affect perceived control in relation to the home (Oswald et al., 2003). The questionnaire was interview-administered, with five response options (1 = strongly disagree to 5 = strongly agree; Oswald et al., 2006). A psychometrically evaluated PD version was used, consisting of 14 items and a total sum score from 14 to 70 (higher scores = higher external control beliefs; Andersson et al., 2020). For participants with only one or two missing answers on item level (*n* = 11, i.e., 7.1% at T2), individual imputation was implemented, using the mean of the recorded values to impute missing item scores and calculate a total sum score.

### Activities of Daily Living

Difficulties in ADL were assessed with the self-reported PD-specific single-item Parkinson’s Disease Activities of Daily Living Scale, PADLS (Hobson et al., 2001; Jonasson et al., 2017). PADLS has five response options with detailed descriptions, summarized as follows: 1 = no difficulties, 2 = mild difficulties, 3 = moderate difficulties, 4 = high levels of difficulties, 5 = extreme difficulties in ADL. A dichotomized version used in other studies (Jonasson et al., 2017; Lindholm et al., 2014) was used when exploring hypothesis 2. PADLS has been psychometrically evaluated for people with PD. One study reported good test-retest reliability (Hobson et al., 2001). Moreover, PADLS scores have been shown to have satisfactory and acceptable data completeness, targeting, and external construct validity (Hobson et al., 2001; Jonasson et al., 2017). It seems to be well suited as a rough estimate of ADL disability in people with PD.

### Confounders

As previous research has shown that age (Table 1), disease severity, cognitive function, and housing accessibility (Table 2) seem to play a role in the relationship between external HCB and ADL they were used as confounders. Age was included as a continuous variable. The Hoehn and Yahr scale (HY, range I-V, higher score = worse disease severity) was used to assess disease severity in “on-state” (Hoehn & Yahr, 1967). The Montreal Cognitive Assessment (MoCA) was used to assess global cognitive function (scores 0–30, higher score = better cognitive function; Nasreddine et al., 2005).

**Table 2.** Variables Used to Test the Hypotheses in the Parkinson’s Disease Sample (*N* = 154).

Variable	*n* (%) unless stated otherwise	Missing, *n*
Hypothesis 1 (more difficulties in ADL lead to higher external HCB)		
Dependent variable: external HCB, median (q1–q3), T2	34 (27–43)	-
Independent variable: PADLS, median (q1–q3), T1	2 (2–2)	-
1. No difficulties	35 (22.7)	
2. Mild difficulties	87 (56.5)	
3. Moderate difficulties	26 (16.9)	
4. Severe difficulties	5 (3.3)	
5. Extreme difficulties	1 (0.6)	
Hypothesis 2 (higher external HCB lead to more dependence in ADL)		
Dependent variable: PADLS dichotomized, T2		-
No difficulties—mild difficulties (scores 1–2)	104 (67.5)	
Moderate difficulties—extreme difficulties (scores 3–5)	50 (32.5)	
PADLS, median (q1–q3), T2	2 (2–3)	
Independent variable: external HCB, median (q1–q3), T1	30 (25–38)	-
PADLS dichotomized, T1		
No difficulties—mild difficulties (scores 1–2)	122 (79.2)	
Moderate difficulties—extreme difficulties (scores 3–5)	32 (20.8)	
Confounding variables in both hypotheses, T1^a^		
Disease severity (HY during on phase), median (q1–q3), T1	2 (2–3)	-
HY I	35 (22.7)	
HY II	55 (35.7)	
HY III	36 (23.4)	
HY IV	26 (16.9)	
HY V	2 (1.3)	
Global cognitive function (MoCA), median (q1–q3), T1	26 (23–28)	2
RAPS categories, T1		-
Expected number of barriers/no barriers due to no functional limitations	79 (51.3)	
Fewer barriers than expected	42 (27.3)	
More barriers than expected	33 (21.4)	

*Note.* ADL = Activities of Daily Living; HCB = external housing-related control beliefs measured with external Housing-Related Control Beliefs Questionnaire; T2 = 3-year follow-up; PADLS = Parkinson’s Disease Activities of Daily Living Scale; T1 = baseline; HY = Hoehn & Yahr; MoCA = Montreal Cognitive Assessment; RAPS = Relative Accessibility Problem Score.

a Age at T1 was also included as a confounder, for descriptive information see Table 1.

Housing accessibility problems were captured with the Relative Accessibility Problem Score (RAPS), based on the Housing Enabler (HE) instrument (Iwarsson & Slaug, 2010). HE is comprised of separate assessments of a personal (P) component (functional limitations/dependence on mobility devices) and an environmental (E) component (environmental barriers in the home). The P and E components are combined through a scoring matrix (HE score, 0–1,844, higher score = more accessibility problems), which produces a measure of the P × E interaction effect (Iwarsson et al., 2012; Iwarsson & Slaug, 2010). A novel analytic approach was applied to separate the P × E interaction effect from the main effects of P and E (Slaug et al., 2019). Using linear regression analysis with the natural logarithm of P as the independent and the interaction term of P × E as the dependent variable, RAPS was created based on the residuals, with three categories: (1) Expected number of barriers/no barriers due to no functional limitation, (2) Fewer barriers than expected, (3) More barriers than expected. As a cut-off for the categorization, we used the difference from the expected number of barriers in any direction of at least four environmental barriers more or less than expected (Slaug et al., 2019).

## Statistical Analysis

Based on the two hypotheses, one linear and one logistic regression analysis were computed due to the different nature of the dependent variables.

Prior to computing the regression analyses, the relationship between the independent variables (external HCB/PADLS, age, MoCA, HY, and RAPS; all at T1) was evaluated with Spearman’s correlation coefficient (*r_s_*). Correlations > 0.7 were considered as multicollinear (Tabachnick & Fidell, 2013), but all were <0.5.

Linear regression analysis was used with external HCB (T2) as the dependent variable (hypothesis 1). The underlying model assumptions for linear regression analyses (Tabachnick & Fidell, 2013) were fulfilled. Logistic regression analysis was used with PADLS (T2) as the dependent variable (hypothesis 2). The Hosmer and Lemeshow test of goodness of fit (Hosmer et al., 2013) supported this approach.

Both regression analyses were first computed by controlling for the baseline value of the dependent variables (linear regression = external HCB T1/logistic regression = PADLS T1), followed by analysis that controlled for the baseline value of the dependent variables and the confounders (HY, MoCA, RAPS, and age; all at T1).

To evaluate changes over time, McNemar’s test was used for PADLS, and paired samples *t*-test was used for external HCB. All analyses were computed in the IBM SPSS Statistics, version 27, with the level of statistical significance set to *p* < .05.

## Results

At baseline, the median (q1–q3) score for external HCB was 30 (25–38) and increased significantly to 34 (27–43; *p* < .001) at follow-up. The participants who experienced more difficulties in ADL (“moderate/severe/extremely difficulties”) increased significantly (*p* = .006) over the 3 years studied, from 32 (20.8%) at baseline to 50 (32.5%) at the follow-up (Table 2).

## Hypothesis 1

**Figure 1.**
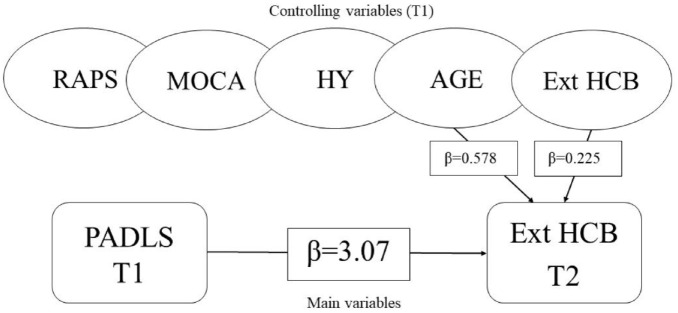
Linear regression analysis supporting hypothesis 1 among people with Parkinson’s disease. *Note.* RAPS = Relative housing Accessibility Problems Scores; MOCA = Montreal Cognitive Assessment; HY = Hoehn & Yahr; Ext HCB = external Housing-Related Control Beliefs Questionnaire; PADLS = Parkinson’s Disease Activities of Daily Living Scale; T1 = baseline; = significant (*p* < .05) impact external HCB; T2 = 3-year follow-up.

In the linear regression analysis controlled for external HCB at T1, there was a statistically significant (*p* < .001) independent effect between external PADLS and HCB (Table 3). Controlling for age, HY, MoCA, and RAPS, there was a statistically independent effect between external PADLS and HCB (β = 3.07) (Figure 1). That is, a 1-point increase on PADLS (i.e., more ADL difficulties) implies an average 3-point increase on external HCB, thereby supporting the assumption of hypothesis 1.

**Table 3.** Regression Analyses Exploring the Association Between HCB and PADLS Among People With Parkinson’s Disease.

Linear regression analyses, *N* = 152 (Dependent variable: External HCB, T2)				
Hypothesis 1 (more difficulties in ADL lead to higher external HCB)				
Independent variable: PADLS (T1)		Regression coefficient (β)	95% [CI]	*p*-Value
Analysis adjusted for HCB (T1)		2.36	0.63, 4.09	.008
Analysis adjusted for HCB (T1) + confounding factors^a^		3.07	1.28, 4.85	<.001
Logistic regression analyses, *N* = 152 (Dependent variable: PADLS^b^, T2)				
Hypothesis 2 (higher external HCB lead to more difficulties in ADL)				
Independent variable: External HCB (T1)		OR	95% [CI]	*p*-Value
Analysis adjusted for PADLS (T1)		1.05	1.01, 1.10	.021
Analysis adjusted for PADLS (T1) + confounding factors^a^		1.04	0.99, 1.09	.136

*Note.* HCB = external Housing-Related Control Belief Questionnaire; T2 = 3-year follow-up; PADLS = Parkinson’s Disease Activities of Daily Living Scale; T1 = baseline; Regression coefficient (β) = unstandardized beta coefficient; OR = Odds Ratio; CI = Confidence Interval.

a Adjusted with the following variables at T1: age, relative housing accessibility problems scores (RAPS), disease severity (Hoehn & Yahr) and global cognitive functioning (Montreal Cognitive Assessment).

b Dichotomization of PADLS: no difficulties—mild difficulties, scores 1 to 2 = 0; moderate difficulties—extreme difficulties scores 3 to 5 = 1.

## Hypothesis 2

In the logistic regression analysis controlled for PADLS at T1, there was a statistically significant independent effect between external HCB and PADLS (*p* = .021). Controlling for additional factors (age, HY, MoCA, and RAPS), no statistically independent effect between external HCB and PADLS could be shown (*p* = .136) (Table 3). Only HY scores were significantly (*p* = .002) associated with external HCB.

## Discussion

This study addresses both directions in the longitudinal relationship between ADL and external housing-related control beliefs, since knowledge is scarce regarding this aspect, not only among people with PD but also in gerontological research on older people in general. The main finding is that difficulties in ADL seem to lead to higher external control beliefs related to housing among people with PD. In contrast, the findings do not support the hypothesis that higher external housing-related control beliefs lead to ADL difficulties. Shedding new light on an insufficiently explored relationship, this knowledge is valuable for theory development on housing and health.

### Findings in Relation to Hypothesis 1: More Difficulties in ADL Lead to Higher External HCB

Our longitudinal findings suggest that more difficulties in ADL can elicit higher external housing-related control beliefs among people with PD. With increasing ADL difficulties the person becomes more dependent on external support, for example, assistance from family members and home care services (Rosqvist et al., 2021). Our finding is in line with a previous cross-sectional study based on the HHPD project, which suggested that people with PD who have more difficulties and dependence in ADL tend to rely on external influences for managing their housing situation (Nilsson et al., 2016). Our current finding also aligns well with clinical reasoning in relation to the field of PD. With a neurodegenerative disease like PD (Váradi, 2020), it is not surprising that a person experiencing increased difficulties in ADL perceives higher external housing-related control beliefs, such as relying on others. Further studies are needed to verify whether the current finding is disease specific, but it indicates the importance of targeting ADL if the aim is to influence external HCB in people with PD.

The results also indicate that both external housing-related control beliefs and PADLS increase significantly over time, which is in line with the previous literature (Chaudhury & Oswald, 2019; Sperens et al., 2020). Such basic knowledge is valuable and can benefit future PD studies on housing and health.

### Findings in Relation to Hypothesis 2: Higher External HCB Lead to More Difficulties in ADL

When all confounding factors are considered, our longitudinal findings suggest that higher external housing-related control beliefs do not lead to difficulties in ADL among people with PD. We chose to base our hypotheses on existing theoretical gerontological frameworks that the current findings do not confirm. However, we are not surprised, as our previous cross-sectional PD study indicated this (Nilsson et al., 2016). A previous longitudinal PD study (based on the HHPD project) included both ADL and external HCB (Gefenaite, Björk et al., 2020). It aimed to assess the association between housing accessibility and ADL, where HCB was shown to play a moderating role. HCB does seem to play a role in these complex relationships, but so does general self-efficacy (Gefenaite, Björk et al., 2020). Among older people in general, we have failed to identify any longitudinal study that has investigated the directions of the relationship between HCB and ADL.

### Findings in Relation to Existing Gerontology Frameworks

Our findings contrast with the assumptions in existing gerontology frameworks and literature that address older people in general, which indicate that external HCB lead to health problems over time (Chaudhury & Oswald, 2019; Oswald et al., 2006; Wahl et al., 2012). Although empirical studies have found cross-sectional associations between external housing-related control beliefs and ADL dependence among older people in general (Iwarsson et al., 2007; Oswald, Wahl, Schilling, & Iwarsson, 2007; Tomsone et al., 2013), none targeted the direction of the relationship. However, such results have informed existing theoretical frameworks, where housing-related control beliefs are assumed to affect health. For example, Chaudhury & Oswald’s (2019) “Integrative conceptual framework of person-environment exchange” assumes that processes that include control beliefs (agency) affect autonomy (including ADL performance). People with PD have characteristic symptoms, such as bradykinesia and postural instability, often in combination with cognitive decline and fatigue (Váradi, 2020). These symptoms can alter prerequisites for ADL performance and lead to a more external locus of control (Sjödahl Hammarlund et al., 2018). Therefore, the relationship between external HCB and ADL difficulties is likely to differ from the relationship in the general aging population, and might change over time because of the progressive nature of the disease. This could explain why our results contradict existing theory and indicate that housing and health dynamics are specific for people with PD.

## Methodological Considerations

A strength of this study is that it addresses an obvious knowledge gap (Iwarsson et al., 2022), and that we use instruments that are psychometrically evaluated for use among people with PD. The external HCB Questionnaire (Oswald et al., 2003) was developed to target the general aging population, founded on psychological theories (Low & Altman, 1992). The questionnaire originally consisted of two sub-scales (powerful others and chance, faith or luck) and has been psychometrically evaluated for use with different sub-groups of the general aging population (Boonyaratana et al., 2021; Oswald et al., 2003). However, a psychometric evaluation in a PD sample suggested that the two sub-scales should be used as one (Andersson et al., 2020). A detail to keep in mind is that we used 1 point to indicate a change, while according to our psychometric evaluation a change should be at least 5 points in any direction to exceed the measurement error (Andersson et al., 2020).

PADLS is a rough measure for evaluation of ADL difficulties, but was developed to capture PD-specific ADL and has been psychometrically evaluated with satisfactory results (Hobson et al., 2001; Jonasson et al., 2017). The fact that PD symptoms fluctuate throughout the day or even by the hour (Váradi, 2020) implies that self-rated ADL difficulties over the previous 4 weeks appear more relevant than a one-time assessment by a health care professional. Another strength is that we trained all data collectors to reduce the risk of introducing a bias.

Patient-reported outcomes might be afflicted with a risk of bias (e.g., recall bias), but we argue that all instruments and assessments have pros and cons. While we adjusted our analyses for confounding factors, there might be additional variables that impact both the independent and dependent variables, such as depressive symptoms, general self-efficacy, or social factors. We recommend future studies based on larger samples as well as studies with more follow-ups, which would allow for addressing more confounding factors. Overall, the interaction of health and housing is complex and warrants more attention to improve understanding.

The 11 individuals excluded due to missing data were older, had more motor symptoms, and increased disease severity (data not shown) than those included. This indicates that the external HCB might not be suitable for those with advanced PD, and the exclusion of these participants affects the external validity of the findings. Reasoning further about external validity, the study sample included few individuals with extreme difficulties in ADL and severe PD, and the results should be interpreted with this in mind. Whether our findings apply for different subtypes of PD remain to be shown in future studies.

A drawback of this study is that different regression analyses had to be used for the two hypotheses because of the different nature of the two dependent variables, which hampers comparisons of the results. We did consider ordinal regression analysis to explore hypothesis 2, but the assumptions for ordinal regression were not met (Armstrong & Sloan, 1989) and the sample size was at the lower end, logistic regression analysis was used. The findings of this exploratory study should be interpreted with caution and mainly used as inspiration for future research.

## Conclusions

The findings suggest that the direction of the relationship is such that difficulties in ADL lead to changes in HCB among people with PD, rather than the other way around. This is contrary to environmental gerontology theories, where control beliefs are assumed to be the driving factor in the relationship. This new knowledge adds to the understanding of housing and health interactions among people with PD, and can be useful for theory development in environmental gerontology in general as well as for this specific sub-group of the aging population. Although other studies need to verify whether these findings are disease specific, our findings indicate the importance of targeting ADL if the ambition is to influence perceived aspects of home in people with PD.
